# Major viral diseases in grain legumes: designing disease resistant legumes from plant breeding and OMICS integration

**DOI:** 10.3389/fpls.2023.1183505

**Published:** 2023-05-09

**Authors:** Uday Chand Jha, Harsh Nayyar, Anirudha Chattopadhyay, Radha Beena, Ajaz A. Lone, Yogesh Dashrath Naik, Mahendar Thudi, Pagadala Venkata Vara Prasad, Sanjeev Gupta, Girish Prasad Dixit, Kadambot H. M. Siddique

**Affiliations:** ^1^ Indian Institute of Pulses Research (IIPR), Indian Council of Agricultural Research (ICAR), Kanpur, Uttar Pradesh, India; ^2^ Department of Botany, Panjab University, Chandigarh, India; ^3^ Department of Plant Pathology, Pulse Research Station, S.D. Agricultural University SK Nagar, SK Nagar, Gujarat, India; ^4^ Department of Plant Physiology, College of Agriculture, Vellayani, Kerala Agricultural University (KAU), Thiruvananthapuram, Kerala, India; ^5^ Dryland Agriculture Research Station, Sher-e-Kashmir University of Agricultural Sciences and Technology (SKUAST)-Kashmir, Srinagar, India; ^6^ Department of Agricultural Biotechnology and Molecular Biology, Dr. Rajendra Prasad Central Agricultural University, Samatipur, Bihar, India; ^7^ Shandong Academy of Agricultural Sciences, Jinan, Shandong, China; ^8^ Center for Crop Health, University of Southern Queensland, Toowoomba, QLD, Australia; ^9^ Department of Agronomy, Kansas State University, Manhattan, KS, United States; ^10^ Indian Council of Agricultural Research, New Delhi, India; ^11^ The University of Western Australia (UWA) Institute of Agriculture, The University of Western Australia, Perth, WA, Australia

**Keywords:** Grain legume, disease, virus, QTL, genome sequence, PTI, ETI

## Abstract

Grain legumes play a crucial role in human nutrition and as a staple crop for low-income farmers in developing and underdeveloped nations, contributing to overall food security and agroecosystem services. Viral diseases are major biotic stresses that severely challenge global grain legume production. In this review, we discuss how exploring naturally resistant grain legume genotypes within germplasm, landraces, and crop wild relatives could be used as promising, economically viable, and eco-environmentally friendly solution to reduce yield losses. Studies based on Mendelian and classical genetics have enhanced our understanding of key genetic determinants that govern resistance to various viral diseases in grain legumes. Recent advances in molecular marker technology and genomic resources have enabled us to identify genomic regions controlling viral disease resistance in various grain legumes using techniques such as QTL mapping, genome-wide association studies, whole-genome resequencing, pangenome and ‘omics’ approaches. These comprehensive genomic resources have expedited the adoption of genomics-assisted breeding for developing virus-resistant grain legumes. Concurrently, progress in functional genomics, especially transcriptomics, has helped unravel underlying candidate gene(s) and their roles in viral disease resistance in legumes. This review also examines the progress in genetic engineering-based strategies, including RNA interference, and the potential of synthetic biology techniques, such as synthetic promoters and synthetic transcription factors, for creating viral-resistant grain legumes. It also elaborates on the prospects and limitations of cutting-edge breeding technologies and emerging biotechnological tools (e.g., genomic selection, rapid generation advances, and CRISPR/Cas9-based genome editing tool) in developing virus-disease-resistant grain legumes to ensure global food security.

## Introduction

Grain legumes, rich in essential amino acids, vitamins, and minerals, are a crucial component of agroecosystems and vital for combating protein- and micronutrient-related malnutrition problems in the growing human population (Graham and Vance, 2003; Jha et al., 2022a). However, changing global climate trends have increased the incidence of various biotic and abiotic stresses, including viral diseases that cause significant yield losses in grain legumes worldwide. It has been estimated that plant viral diseases alone cause 50% of plant disease globally with economic losses measuring $30 billion annually (Hilaire et al., 2022). To address this challenge, researchers are developing sustainable and ecofriendly approaches to design the next generation of virus-disease-resistant grain legumes. Plants have sophisticated innate immune systems that respond to attacking viral pathogens using pattern-triggered immunity (PTI) and effector-triggered immunity (ETI) (Jones and Dangl, 2006). Mendelian-based genetic approaches initially helped uncover the major genetic determinants controlling these diseases in various legumes. Subsequently, molecular marker technologies facilitated the identification of causative virus-disease-resistant genomic regions/QTL in various grain legumes using biparental QTL analysis and genome-wide association studies (GWAS). Decoding various grain legume genome sequences enabled the discovery of novel single nucleotide sequence (SNP) markers for GWAS to explore marker-trait associations/haplotypes for disease resistance at the whole-genome level (for details see Ha and Lee, 2020). Whole-genome resequencing and the availability of pangenomes in various legumes and viruses offer novel opportunities to explore presence/absence variations, structural variations conferring viral disease resistance, and novel resistance (R) gene(s) and virulence gene(s) across the whole genome (Zhao et al., 2020; Jha et al., 2022b). These approaches also offer novel insights into the various effector molecules of virulent viruses to design virus-disease-resistant grain legumes. In addition, advances in functional genomics using RNA-seq-based transcriptome analysis technologies have enriched our understanding of possible candidate genes and regulatory non-coding RNAs attributing to resistance against various virus diseases with putative functions. In recent years, researchers have made progress in designing virus-resistant grain legumes using genetic-engineering-driven approaches, such as RNA interference (RNAi) and virus-induced gene silencing (VIGS) (Cruz and Aragão, 2014; Gao et al., 2015; Xun et al., 2019; Gao et al., 2020). Moreover, the benefits of emerging novel breeding approaches, such as rapid generation advancement protocol, genomic selection, and genome editing tools, could be harnessed for developing virus-resistant grain legumes. Thus, amalgamation of various ‘omics’ technologies with various novel breeding approaches could greatly benefit us designing future grain legumes cultivars with improved virus resistance (Weckwerth et al., 2020; Vahabi and Michailidis, 2022).

## Viral diseases in soybean (*Glycine max*)

Soybean mosaic virus (SMV; genus *Potyvirus*, family *Potyviridae*) is one of the most devastating seed-borne viral diseases, causing severe yield and quality losses annually (Cho et al., 1977; Ma et al., 2002). As a vector, SMV is transmitted by soybean aphids (*Aphis glycines*) (Wang et al., 2006a). SMV also acts synergistically with bean pod mottle virus (BPMV) (genus *Comovirus*, family *Comoviridae*), decreasing yields by up to 85% (Ross, 1968). SMV virus particles contain a linear, positive sense, single-stranded RNA ~9.6 kb in length (Tolin, 1999). Cho and Goodman (1979); Cho and Goodman (1982) first established a classification system for grouping SMV isolates, reporting seven strain groups (G1–G7) based on the differential reactions of soybean cultivars conferring resistance to a common strain. In Japan, five main strains (A–E) of SMV isolates have been reported (Takahashi et al., 1963; Takahashi et al., 1980). In other studies, SMV was classified into 11 groups in South Korea (Cho et al., 1983; Seo et al., 2009) and 22 groups (SC1–SC22) in China (Guo et al., 2005; Moon et al., 2009; Li et al., 2010; Wang et al., 2013; for details see Usovsky et al., 2022). Stunted growth, mosaic leaf pattern, leaf curling, and seed coat mottling are the major symptoms of SMV (Bowers and Goodman, 1979; Tu, 1992) (see **Table 1**). Early mosaic symptoms appear 5–7 days after infection, and late mosaic symptoms appear 2–3 weeks after infection (Hill, 1999). Tobacco ringspot virus (TRSV), a single-stranded bipartite RNA virus, causes significant yield losses (25–100%) in soybean (Crittenden et al., 1966; Hartman and Domier, 2015), with stunted plant growth, dwarf and rolled leaflets, brownish, necrotic, and brittle buds, and bud death the major symptoms (Crittenden et al., 1966).

**Table 1:** List of major viral diseases, their symptoms and yield losses in major grain legume crops.

Host plant	Virus species	Genus and Family	Genome	Symptoms and losses
Common bean	*Alfalfa mosaic virus*	*Alfamovirus, Bromoviridae*	ssRNA	Mosaic symptoms, scattered bright yellow dots on leaves, deformed beans (Kaiser and Hannan, 1983)
	*Bean common mosaic virus*	*Potyvirus, Potyviridae*	ssRNA	Mosaic, dwarfing, chlorosis, leaf curling (Flores-Estévez et al., 2003)
	*Bean dwarf mosaic virus*	*Begomovirus,Geminiviridae*	ssDNA	Severe stunting and dwarfing, aborted flowers, distorted pods (Wilcox and Laviolette, 1968; Levy and Tzfira, 2010)
	*Bean golden yellow mosaic virus*	*Begomovirus,Geminiviridae*	ssDNA	Epidemics cause up to 100% crop losses (Gálvez and Morales, 1989; Morales and Anderson, 2001)
	*Bean yellow mosaic virus*	*Potyvirus, Potyviridae*	ssRNA	Leaf mosaic symptoms and 30–40% yield losses (Swenson, 1968; Hagedorn and Inglis, 1986)
	*Clover yellow vein virus*	*Potyvirus, Potyviridae*	ssRNA	Yellow mosaic, malformation and reduced plant size (Tracy et al., 1992)
	*Tomato yellow leaf curl virus*	*Begomovirus,Geminiviridae*	ssDNA	Leaf thickening and crumpling (Hedesh et al., 2011)
Cowpea	*Blackeye cowpea mosaic virus*	*Potyvirus, Potyviridae*	ssRNA	Reduced stem height and aboveground fresh weight (Fajinmi, 2019)
	*Cowpea golden mosaic virus*	*Begomovirus*,*Geminiviridae*	ssDNA	Causes mosaic, mottling, necrosis, and stunting, ultimately affecting seed production (Boukar et al., 2013)
	*Cowpea severe mosaic virus*	*Potyvirus, Potyviridae*	ssRNA	Severe mottling of newly emerging leaves and, in severe cases, stunting (Umaharan et al., 1997b)
	*Cucumber mosaic virus*	*Cucumovirus, Bromoviridae*	ssRNA	Chlorosis, vein clearing, necrosis, deformed leaves, mild to severe mosaic and mottle symptoms (Hughes et al., 2003)
Chickpea	*Cucumber mosaic virus* *Chickpea chlorotic stunt virus* *Chickpea chlorotic dwarf virus* *Alfalfa mosaic virus*	*Cucumovirus,Bromoviridae* *Polerovirus, Solemoviridae* *Mastrevirus, Geminiviridae* *Alfamovirus, Bromoviridae*	ssRNA ssRNA ssDNA ssRNA	CMV symptoms occur on shoots, pods, and seeds (Jones et al., 2008) Small leaves, discoloration and bushy stunted appearance are the major symptom of this disease (Kanakala and Kuria, 2018) Chlorosis of the terminal bud followed by necrosis are the major symptoms (Nene et al., 2012)
Mung bean	Mungbean yellow mosaic India virus	*Begomovirus, Geminiviridae*	ssDNA and β-satellite	Bright yellow mosaic symptoms on infected leaves, with few flowers, yellow-spotted pods with immature and deformed seeds causing up to 100% yield losses (Hussain et al., 2004; Akbar et al., 2019; Malathi and John, 2009; Biswas et al., 2012)
	Mungbean yellow mosaic virus	*Begomovirus, Geminiviridae*	ssDNA	Bright yellow mosaic symptoms on leaves, with few flowers (Hussain et al., 2004; Akbar et al., 2019). Pods become yellow-spotted with deformed seed (Malathi and John, 2009).
Pigeonpea	*Pigeon pea sterility mosaic virus*	*Emaravirus, Fimoviridae*	ssRNA	Annual yield losses >US$300 million in India, stunted and bushy plants, smaller leaves with chlorotic rings or mosaic symptoms and no reproductive part formation (Patil and Kumar, 2015).
Soybean	*Soybean mosaic virus*	*Potyvirus, Potyviridae*	ssRNA	Mosaic, dark green leaf areas with misshapen, discolored seed coat, stunted leaflets, necrosis (Gardner and Kendrick, 1921)
Urd bean	*Urdbean leaf crinkle virus*	Uncharacterized	–	Leaf crinkling, curling, puckering and rugosity, stunted plant growth, deformed floral parts (Nene, 1972).

## Viral diseases in common bean (*Phaseolus vulgaris*)

Major viral diseases of common bean resulting in significant yield losses include bean common mosaic virus (BCMV), bean common mosaic necrosis virus (BCMNV), bean yellow mosaic virus (BYMV), clover yellow vein virus (ClYVV), cowpea aphid-borne mosaic virus (CABMV), and watermelon mosaic virus-2 (WMV-2), with ssRNA belonging to family *Potyviridae* and genus *Potyvirus* (for details see Meziadi et al., 2017), and bean golden yellow mosaic virus (BGYMV) and bean dwarf mosaic virus (BDMV), with ssDNA belonging to family *Geminiviridae* and genus *Begomovirus* (Seo et al., 2004). Eight pathogenicity groups in the BCMV complex have been reported (Drijfhout et al., 1978; Drijfhout and Morales, 2005; Feng et al., 2015). BCMV is predominant worldwide in all legume-cultivation areas (Drijfhout, 1978; McKern et al., 1992; Makkouk et al., 2012); however, BCMNV is restricted to Africa, Europe, and North and South America (Worrall et al., 2015). Yield losses due to BCMV and BCMNV infection range from 6–98% (Hagedorn and Inglis, 1986; Worrall et al., 2015). BCMV and BCMNV show similar symptoms, including mosaic, dwarfing, chlorosis, and leaf curling (Flores-Estévez et al., 2003). BYMV, another viral disease of common bean, produces leaf mosaic symptoms and 30–40% yield losses (Swenson, 1968; Hagedorn and Inglis, 1986). For CIYVV, the notable symptoms are yellow mosaic, malformation, and reduced plant size (Tracy et al., 1992) (see **Table 1**).

The main viruses in genus *Begomovirus* that cause serious yield losses in common bean are BGYMV, tomato yellow leaf curl virus (TYLCV), and BDMV (Blair and Morales, 2008). The main symptoms of BGYMV are intense yellowing, pod deformation, stunting, and flower or pod abortion, causing 40–100% yield losses (Morales and Anderson, 2001; Morales, 2006; Blair et al., 2007; Subramanya, 2013; Aragão and Faria, 2009). The characteristic symptoms of TYLCV are leaf thickening and crumpling (Hedesh et al., 2011), while those of BDMV are stunted plant growth and leaf mosaic and mottle symptoms (Seo et al., 2004).

## Viral disease in pigeonpea

Pigeonpea sterility mosaic virus (PPSMV) belongs to genus *Emaravirus*, causing sterility mosaic disease in pigeonpea (*Cajanus cajan*), resulting in yield losses >US$300 million in India (Patil and Kumar, 2015). An eriophyid mite *Aceria cajani* transmits the disease (Patil and Kumar, 2015). The visual symptoms include bushy and pale green appearance, mosaic leaf mottling, reduced leaf size, and partial/complete failure or no formation of reproductive structures (Daspute et al., 2014; Patil and Kumar, 2015).

## Viral diseases in mungbean (*Vigna radiata*)

Two species cause yellow mosaic disease—mungbean yellow mosaic virus (MYMV) and mungbean yellow mosaic India virus (MYMIV)—which significantly affect mungbean productivity (Tsai et al., 2013). MYMIV is a major yield constraint in mungbean in South and Southeast Asia (Selvi et al., 2006; Iqbal et al., 2011), causing up to 100% yield losses under congenial conditions (Bashir et al., 2006). MYMV and MYMIV are begomoviruses with genomes comprising circular single-stranded DNA-A and DNA-B components known as ‘legumoviruses’ (Ilyas et al., 2009). This disease is transmitted by white fly (*Bemisia tabaci* Genn.) (Nariani, 1960). The disease features bright yellow mosaic symptoms on infected leaves, with few flowers, yellow-spotted pods, and immature and deformed seeds causing up to 100% yield losses (Hussain et al., 2004; Malathi and John, 2009; Biswas et al., 2012; Akbar et al., 2019) (see **Table 1**).

## Viral diseases in urdbean (*Vigna mungo*)

Yellow mosaic disease (YMD) caused by MYMIV (Mayo, 2005) is the most destructive viral disease of urdbean, causing significant yield losses (Singh, 1980). MYMIV belongs to the group *Geminiviridae* and is transmitted by whitefly (*B. tabaci* Genn.) (Nariani, 1960). The characteristic symptoms of YMD include scattered yellow chlorotic spots on leaves, which enlarge and coalesce, resulting in conspicuous systemic bright patches, with the leaves eventually turning yellow (Kundu and Pal, 2012). Infected plants exhibit stunted growth, delayed maturity, and reduced flower and pod numbers (Grewal, 1978). Urdbean leaf crinkle virus (ULCV) disease decreases yields by 35–81% depending on host genotype and infection condition (Bashir et al., 1991). ULCV is transmitted by seed and sap inoculation, with white fly and aphid insect pests acting as vectors (Beniwal and Bharathan, 1980). The characteristic symptoms of this disease are leaf crinkling, curling, puckering, rugosity, stunted plant growth, and deformed floral parts (Nene, 1972) (see **Table 1**).

## Viral diseases in cowpea (*Vigna unguiculata*)

Twenty viruses have been reported in cowpea (Hampton et al., 1997; Lima et al., 2005); however, the major viruses causing yield limitations are (1) cowpea severe mosaic virus (CPSMV; family *Comoviridae*, genus *Comovirus*); (2) cowpea aphid-borne mosaic virus (CABMV; family *Potyviridae*, genus *Potyvirus*); (3) cucumber mosaic virus (CMV; family *Bromoviridae*, genus *Cucumovirus*); (4) cowpea golden mosaic virus (CGMV; family *Geminiviridae*, genus *Begomovirus*) (Lima et al., 2005). Leaf crinkling and severe mottling of newly emerging leaves are characteristic symptoms of CPSMV, with stunting in severe cases (Umaharan et al., 1997b). CABMV is a seed-borne disease causing 13–87% yield losses under field conditions (Bashir et al., 2002). Virus-infected seed provides the initial inoculum, with aphids contributing secondary spread of the disease (Bashir et al., 2002). The characteristic features of CMV-caused disease in cowpea are chlorosis, vein clearing, necrosis, leaf deformation, and mild to severe mosaic and mottle (Hughes et al., 2003) (see **Table 1**). For CGMV infection, plants show mosaic, mottling, necrosis, and stunting, with low seed production (Boukar et al., 2013).

## Viral diseases in groundnut

The most notable viral diseases in groundnut are peanut bud necrosis virus (PBNV), tobacco streak virus (TSV), peanut mottle virus (PeMoV), and Indian peanut clump virus, causing significant yield losses (Radhakrishnan et al., 2016). PBNV, belonging to genus *Tospovirus*, is transmitted by *Thrips palmi*, causing 30–90% yield losses (Vemana et al., 2015). Necrosis of terminal buds is a characteristic feature of this disease (Radhakrishnan et al., 2016). Peanut stem necrosis disease, caused by TSV, belongs to genus *Ilarvirus* of family *Bromoviridae* (Prasada Rao et al., 2003). Characteristic symptoms of this disease are complete stem necrosis and necrotic lesions on terminal leaflets (Radhakrishnan et al., 2016).

## Viral diseases in chickpea

Chickpea is susceptible to several viral diseases including chickpea stunt disease (CSD). It is an emerging concern, posing a serious challenge to chickpea production and causes up to 95% yield loss (Abraham and Vetten, 2022). Interestingly, two different pathogens, *viz*., *Chickpea chlorotic stunt virus* (CpCSV), a member of Polerovirus (Abraham and Vetten, 2022) and *Chickpea chlorotic dwarf virus* (CpCDV), a member of *Mastrevirus*, family *Geminiviridae* (Kanakala and Kuria, 2018) are found to be associated with this disease. Small leaves, discolotration and bushy stunted appearance are the major symptoms of this disease. Stunt [bean (pea) leaf roll virus] is an important viral disease in chickpea mostly prevalent in chickpea-growing regions across the world. Stunting and phloem browning are the most visible symptom of this disease, the leaflets of infected plants become yellow, brown or orange in color (Nene et al., 2012). Of the other viruses, cucumber mosaic virus (CMV) causes significant yield losses in chickpea, reportedly 45%, when CMV incidence reached 75% (Jones et al., 2008). CMV symptoms occur on shoots, pods, and seeds. CMV also decreases seed quality (Jones et al., 2008). Mosaic [alfalfa mosaic virus] is a minor viral disease primarily found in Algeria, India, Iran, and Morocco, which causes terminal bud chlorosis followed by necrosis (Nene et al., 2012). Necrosis, caused by lettuce necrotic yellow virus, is characterized by twisted main and axillary shoots and necrotic tip burn on leaves (Nene et al., 2012).

## Plant genetic resources for developing virus-resistant legumes

Various approaches, from breeding and plant protection to integrated approaches, have been embraced to develop virus-resistant legume crops (Bag et al., 2014; Meziadi et al., 2017). For example, plant breeding is a viable and sustainable approach for identifying grain legume landraces, accessions, and breeding lines, requiring no chemical pesticides that adversely affect the environment (Jha et al., 2020). Further, equipping grain legume cultivars with virus-resistant gene(s) using pre-breeding approaches, backcross breeding, and other modern breeding tools could help design virus-resistant grain legumes.

Several resistance sources are available for soybean SMV, including PI 96983 (Kiihl and Hartwig, 1979; Chen et al., 1991), ‘Columbia’ (Ma et al., 2002), PI 88788 (Gunduz et al., 2004), L29 (Buss et al., 1999), and PI 486355 (Ma et al., 1995), along with several near-isogenic lines, including L96-1676, L96-1680, L96-1683, and L96-1687 carrying the *Rsv1* resistance gene developed from Williams × Buffalo, V97-9001 and V97-9003 carrying the *Rsv4* resistance gene developed from Essex × PI 486355, and L88-8431 and L88-8440 carrying the *Rsv1-r* resistance gene developed from Williams × Raiden (Wang et al., 2006b; for details, see Usovsky et al., 2022). In addition, soybean genotypes harboring the *Rsv1Rsv3* gene conferring resistance against SMV include ‘Hourei’ (Gunduz et al., 2002), ‘OX670’ (Gunduz et al., 2001), ‘Tousan’ (Gunduz et al., 2002), ‘J05’ (Zheng et al., 2006), ‘Zao18’ (Liao et al., 2002), and ‘Jiodou1’ (Shi et al., 2008) (see **Table 2**). Likewise, the ‘8101’ genotype harboring the *Rsv1Rsv3Rsv4* gene (Liao et al., 2011) could be used to pyramid various genes conferring resistance against SMV. Intending to transfer SMV resistance into high-yielding soybean genotypes, Kato et al. (2016) introgressed genes conferring resistance against Japanese strains SMV-C and D into ‘Fukuibuki’ from ‘Harosoy’ donor parents *via* backcross breeding.

**Table 2:** Grain legume genotypes conferring resistance against various viral diseases.

Crop	Disease	Resistance source	Reference
Common bean	Bean golden mosaic virus	Aete 1/37, Aete 1/38, Aete 1/40, Rosinha G2/69, Carioca 99, 9236-6, 9245-94, MD 806, MD 807, MD 820, MD 829, MD 808, MD 821 and PR9556-171	Pompeu and Kranz (1977); Velez et al. (1998); Bianchini (1999); Román et al. (2004)
	Bean yellow mosaic virus	*P. coccineus* ‘Kelvedon’ ‘Marvel 3120-27’	Schroeder and Provvidenti (1968)
	Bean yellow mosaic virus/clover yellow vein virus	*P. coccineus* ‘B28S2C’	Dickson and Natti (1968); Hart and Griffiths (2015)
	Bean pod mottle virus	‘BAT93’	Thomas and Zaumeyer (1950); Pflieger et al. (2014)
	Bean dwarf mosaic virus	Othello	Seo et al. (2004)
	Mungbean yellow mosaic India virus	Anupam	Patwa et al. (2020)
	Tomato yellow leaf curl virus	‘GG12’	Monci et al. (2005)
	Cucumber mosaic virus	‘Othello’	Seo et al. (2006)
	Bean common mosaic virus and bean common mosaic necrosis virus	‘Black Turtle-1’	Ali (1950); Provvidenti (1974); Drijfhout et al. (1978); Kyle et al. (1986); Kyle and Provvidenti (1987)
Cowpea	Blackeye cowpea mosaic virus	PI 441918, Pinkeye Purple Hull-BVR	Gillaspie (2001)
	Bean common mosaic virus-blackeye cowpea mosaic strain	IT-98 K-1092-1	Ogunsola et al. (2021)
	Cucumber mosaic virus and blackeye cowpea mosaic virus	GC-86L-98	Gillaspie (2002)
	Cowpea mottle carmovirus	*Vigna vexillata*	Ogundiwin et al. (2002)
	Cowpea aphid-borne mosaic virus	Purple Knuckle Hull‐55, MNC‐03‐731C‐21, and CNCx284‐66E	Lima et al. (2011)
	Southern bean mosaic virus	IT97K-1069-6 and IT04K-405-5	Ogunsola et al. (2021)
Mungbean	Urdbean leaf crinkle virus	VC-3960 (A-88), VC-3960 (A- 89), 98-CMH-016, NM-2, and BRM-195	Bashir et al. (2006)
	Mungbean yellow mosaic disease	NM 94, ML 1628	Nair et al. (2017)
	Yellow mosaic disease	AVMU 1698, AVMU 1699, AVMU 16100, AVMU 16101, and KPS 2	Nagaraj et al. (2019)
	Urdbean leaf crinkle virus	RME-16-3, RME-16-12, MLT-GG R-16-007, MLT-GG R-16-009, and COGG 1319	Sravika et al. (2019)
Pigeon pea	Pigeon pea sterility mosaic	ICPL 7035, ICP 15614, 15615, 15626, 15684, 15688, 15700, 15701, 15725, 15734, 15736, 15737, 15740,15924, 15925, 15926, ICPs 6739, 8860, 11015, 13304, and 14819	Saxena et al. (2004); Kumar et al. (2005); Sharma et al. (2012)
Soybean	Soybean mosaic virus	PI96983, L29, V94-5152, PI486355, V94-5152, Columbia, Raiden soybean (PI 360844), PI 507389, PI 96983, and PI 88788	Kiihl and Hartwig (1979); Buss et al. (Buss et al., 1997; Buss et al., 1999); Ma et al. (Ma et al., 1995; Ma et al., 2002; Ma et al., 2003); Hayes et al. (2000); Chen et al. (2002); Gunduz et al. (2004); Wen et al. (2013)
	Yellow mosaic virus	PS19, JS9752, PK564, RKS18, Kalitur, RAUS5, PK1042, PS1241, Shilajeet, MAUS71, PK1024, PK416, Alankar, Bragg, Ankur, and PK262	Das et al. (2017)
Urdbean	Urdbean leaf crinkle virus	2cm-703, 90cm-015, 93cm-006, 94cm-019, 99cm-001, IAM 382-1, IAM382-9, IAM382-15, and IAM133	Ashfaq et al. (2007)
	Mungbean yellow mosaic disease	Pant U-84, UPU-2, IC144901, IC001572, IC011613, and IC485638	Singh (1980); Verma and Singh (1986); Bag et al. (2014)
	Urdbean leaf crinkle virus	M-6206, IAM-382-15, IAM-133, and Mash-1	Binyamin et al. (2011)
	Mungbean yellow mosaic India virus	VMR 84	Kundu and Pal (2012)

The common bean genotype ‘Redlands Greenleaf C’ harbors the *bc-1* resistance gene against BCMV (Drijfhout, 1978; Strausbaugh et al., 1999; Miklas et al., 2000). Other soybean genotypes confer resistance against combined BCMV/BCMNV, including ‘Olathe’ harboring the *bc-u* gene (Drijfhout, 1978; Strausbaugh et al., 1999; Miklas et al., 2000), ‘Nodak’ harboring the *bc-12* gene (Drijfhout, 1978; Miklas et al., 2000), Michelite’ harboring the *bc-2* gene (Drijfhout, 1978; Miklas et al., 2000), ‘N85120’ harboring the *bc-22* gene (Drijfhout, 1978; Kelly et al., 1995), and ‘B85009’ harboring the *bc-3* gene (Drijfhout, 1978; Mukeshimana et al., 2005). In addition, Hart and Griffiths (2013) reported that ‘Clipper’ and ‘Jolanda’ soybean genotypes harbor the *cyv* and *desc* genes, respectively, conferring resistance against ClYVV (see **Table 2**), while ‘A429’ or ‘9236-6’ harboring the *bgm-1* gene (Urrea et al., 1996; Blair et al., 2007) and ‘DOR303’ harboring the *bgm-2* gene (Velez et al., 1998) confer resistance against BGYMV.

A multilocation evaluation of mungbean genotypes across India explored the resistance source against MYMV. The mungbean line ‘NM 94’ was identified as resistant against MYMV in the eastern state of Odisha but only moderately resistant in the southern state of Tamil Nadu. The evaluation identified ‘ML 1628’ as a source of high resistance against MYMV (Nair et al., 2017). Basavaraj et al. (2019) screened 14 mungbean genotypes over three seasons for MYMV resistance, reporting five genotypes (AVMU 1698, AVMU 1699, AVMU 16100, AVMU 16101, and KPS) with resistance. A rigorous field screening of a diverse set of 344 urdbean genotypes was tested for YMV resistance under field conditions for two years (Bag et al., 2014). Eight resistant genotypes were tested further in a glasshouse, identifying IC144901 and IC001572 as highly resistant against YMV (Bag et al., 2014).

Ashfaq et al. (2007) screened 87 urdbean genotypes for ULCV resistance over two seasons under field conditions. Based on the disease severity index, nine genotypes (2cm-703, 90cm-015, 93cm-006, 94cm-019, 99cm-001, IAM 382-1, IAM382-9, IAM382-15, and IAM133) were highly resistant to this disease. Likewise, a field screening of 40 urdbean genotypes identified M-6206, IAM-382-15, IAM-133, and Mash-1 as ULCV resistant (Binyamin et al., 2011). Sravika et al. (2019) conducted a field screening of 107 mungbean genotypes, reporting RME-16-3, RME-16-12, MLT-GG R-16-007, and MLT-GG R-16-009 as highly resistant to ULCV in *rabi*-sown mungbean but no resistant genotypes in *kharif*-sown mungbean. Cowpea genotypes with resistance against blackeye cowpea mosaic virus and CABMV include TVu401, Tvu1453, and Tvu1948, and advanced breeding lines IT82D-885, IT28D-889, and IT82E-60 (Gumedzoe et al., 1998) (see **Table 2**).

Besides cultivated plant species, crop wild relatives (CWRs) are a valuable source of novel genes associated with biotic and abiotic stresses. Kumar et al. (2005) reported sterility mosaic disease (SMD) resistance in several pigeonpea accessions belonging to six CWRs, including *C. albicans, C. platycarpus, C. cajanifolius, C. lineatus, C. scarabaeoides*, and *C. sericeus.* Among them, 15 accessions, including ICP 15614, 15615, 15626, 15684, 15688, 15700, and 15701, had SMD resistance. Mallikarjuna et al. (2011) reported that lines derived from *C. acutifolius* and *C. platycarpus* exhibited resistance against pigeonpea SMD under field conditions. Similarly, resistance to MYMV was found in *V. radiata* var. *sublobata* Roxb. Verde., a progenitor of mungbean, with resistance genes transferred to commercial mungbean cultivars (Singh and Ahuja, 1977). In chickpea, *C. echinospermum* and other chickpea CWRs (Kahraman et al., 2017; Rajpal et al., 2023) have the potential to transfer viral disease resistance to cultivated species.

## Genetics of viral disease resistance in legumes

Mendelian genetics provides preliminary information on the genetic resistance of viral diseases in grain legumes. Kiihl and Hartwig (1979) developed the gene symbol for controlling resistance against SMV-1 in soybean as *Rsv Rsv* (resistance)*, rsv* ^t^ *rsv* ^t^ (partial resistance), and *rsv rsv* (susceptible). Eight allelic-dominant genes (*Rsv1*, *Rsv1-y*, *Rsv1-m*, *Rsv1-t*, *Rsv1-k*, *Rsv1-s*, *Rsv1-r*, and *Rsv1-h*) for SMV resistance have been reported at the most common locus, *Rsv1* (Chen et al., 1991; Chen et al., 1994; Ma et al., 1995; Chen et al., 2001; Chen et al., 2002). The *Rsv1* gene identified in PI 96983 was mapped to the soybean molecular linkage group ‘F’ (Yu et al., 1994; Yu et al., 1996). The *Rsv2* gene derived from the Raiden cultivar and allelic to the *Rsv1* locus was assigned a new gene symbol *Rsv1-r* (Chen et al., 2002). Subsequently, a new resistant gene *Rsv3*, independent of *Rsv1* and *Rsv2*, was reported (Buzzell and Tu, 1989) and mapped to the molecular linkage group ‘B2’ (Jeong et al., 2002). Ma et al. (2002) shed further light on the genetic resistance of SMV, confirming the presence of two independent resistant *R3* and *R4* genes working in a complementary fashion in the soybean genotype ‘Columbia’. A new resistance gene independent of *Rsv1* and *Rsv3* was reported in PI 486355 and mapped on ‘D1b’ (Hayes et al., 2000). The presence of *Rsv1* and *Rsv3* in ‘OX670’ soybean cultivar conferring resistance to SMV-G1 through G7 was reported (Gunduz et al., 2001). Gunduz et al. (2004) identified resistance against SMV strains G1 and G7. A genetic analysis of resistance in PI 88788 revealed that a single, partially dominant gene controlled SMV-G1; however, the same gene was dominant for SMV-G7 (Gunduz et al., 2004). The authors also confirmed that the resistance gene in PI 88788 was independent of *Rsv1* and *Rsv3*, and the dominant resistance gene in PI 88788 was allelic to the SMV-resistant gene at the *Rsv4* locus in V94-5152. The *Rsv4* gene contributed resistance to all SMV strains (SMV-G1 to SMV-G7) (Chen et al., 1993; Ma et al., 1995). Recently, Klepadlo et al. (2017) assigned a new gene symbol *Rsv5* to the resistance gene in ‘York’ to substitute the old allele named *Rsv1-y* on chromosome 13. Subsequently, several other researchers advocated that a single dominant gene governed the genetic control of SMV resistance (Zheng et al., 2014; Ma et al., 2016; Karthikeyan et al., 2017; Rui et al., 2017; Karthikeyan et al., 2018; Wu et al., 2019; Jin et al., 2022; Liu et al., 2022a).

The inheritance of dominant resistance gene *I* controlling BCMV has been reported in common bean (Ali, 1950; Kyle et al., 1986), BCMNV resistance controlled by the *I* gene (Provvidenti, 1974), watermelon mosaic virus-2 resistance controlled by the *Hsw* and *Wmv* genes (Provvidenti, 1974), and BYMV controlled by the *By-1* gene (Schroeder and Provvidenti, 1968). In contrast, resistance against BCMV and BCMNV is controlled by recessive *bc-1*, *bc-u*, *bc-1^2^*, *bc-2*, *bc-2^2^*, and *bc-3* (Kelly et al., 1995; Strausbaugh et al., 1999; Miklas et al., 2000; Mukeshimana et al., 2005). Similarly, *bgm-1* and *bgm-2* recessive genes (Urrea et al., 1996; Velez et al., 1998) governed resistance against BGYMV and *cyv* and *desc* recessive genes (Hart and Griffiths, 2013) controlled ClYVV resistance. Monogenic (*Bdm*) and dominant resistance of BDMV were established by Seo et al. (2004) using disease reaction data from F_1_, F_2_, F_3_, and reciprocal crosses developed from Othello and Topcrop common bean genotypes.

Singh et al. (1983); Sharma et al. (1984), and Srinivas et al. (1997a); Srinivas et al. (1997b) offered initial insights into the genetic inheritance of SMD resistance in pigeonpea. An evaluation of F_1_ and F_2_ progenies developed from ICP 7035, ICP 7349, and ICP 8850 (resistant) parents and ICP 8863 (susceptible) parent crosses confirmed monogenic inheritance of disease resistance of two SMD isolates (Srinivas et al., 1997a). Subsequently, Nagaraj and Kulkarni (2004) reported that two genes governed SMD resistance based on F_1_, F_2_, BC_1_, and BC_2_ populations developed from crossing resistant (ICP 7035 and MAL 14) and susceptible (TTB 7, ICP 8863, and DBN1) parents. Likewise, Daspute et al. (2014) reported that two separate genes (SV1 and SV2) with inhibitory gene action controlled SMD inheritance.

Recessive inheritance of MYMIV resistance in mungbean has been reported (Khattak et al., 2000; Dhole and Reddy, 2012). Six crosses developed from resistant and susceptible parents and F_1_ and F_2_ populations revealed that two genes controlled MYMIV resistance, one of which was recessive (Dhole and Reddy, 2012).

Genetics resistance of MYMV in blackgram was analyzed in F_1_, F_2_, and backcross populations developed from Pant U-84 × UL-2 and UPU-2 × UL-2 (Singh, 1980), with the results indicating recessive and digenic resistance. The same resistance gene was validated against MYMV by analyzing F_1_, F_2_, and F_3_ developed from Pant U84 and UPU 2 resistant donors (Verma and Singh, 1986). Subsequently, Basak et al. (2005) reported recessive monogenic resistance of YMV based on phenotyping F_1_, F_2_, and F_3_ progenies derived from MYMV-tolerant T-9 × MYMV-susceptible T-9 genotype in response to the YMV reaction.

## Molecular mechanisms involved in plant virus resistance

During viral pathogen attack, plants recruit two branches of the immune defense system to evade the pathogen-associated attack: molecular pattern-triggered immunity (PTI) and effector-triggered immunity (ETI) (Jones and Dangl, 2006). For PTI, pattern recognition receptors (PRRs) on the cell surface of host plants perceive the pathogen-associated molecular patterns (PAMPs) to initiate defense responses known as PRR-triggered immunity (Dodds and Rathjen, 2010; Thomma et al., 2011; Win et al., 2012; Jones et al., 2016; Langner et al., 2018). For ETI, intracellular nucleotide-binding domain and leucine-rich repeat-containing (NB-LRR) encoded by host disease resistance (R) genes inside cells recognize the viral effector, mediating viral growth and disease in host plants (Jones and Dangl, 2006) and initiating the NLR-mediated response known as ETI (vanderHoorn and Kamoun, 2008; Win et al., 2012; Langner et al., 2018). In PTI, following PAMP perception of the viral pathogen through host plant PRRs, plants mediate an influx of extracellular Ca^2+^ in the cytosol, activating downstream immune responses (Ranf et al., 2011; Nomura et al., 2012; Bigeard et al., 2015; Jiang and Ding 2022), inducing reactive oxygen species (Chinchilla et al., 2007; Zhang et al., 2007), and activating various MAP kinase cascades (Zipfel et al., 2006; Bethke et al., 2012) that initiate transcriptional reprogramming of various TFs, such as WRKYs (Mao et al., 2011; Zhang et al., 2012) and downstream disease resistance gene(s). Viral pathogens deploy effector molecules to overcome host-deployed PTI. Once the host plant nucleotide-binding (NB) and leucine-rich-repeat (LRR)-containing receptors recognize these effectors, plants initiate ETI (Jones and Dangl, 2006; Peng et al., 2018; Alhoraibi et al., 2019). The recognition of pathogenic effectors by host NLRs triggers a hypersensitive response that mediates programmed cell death (Gao et al., 2017). The complex molecular mechanisms of plant resistance against viral disease resistance remain elusive. Thus, further investigations are needed to decipher the complete circuit networks and plant signaling pathways involved in response to viral pathogen attack and the pathways and mechanisms of plant immune response.

## QTL mapping for various virus-disease-resistant grain legumes

**Figure 1:**
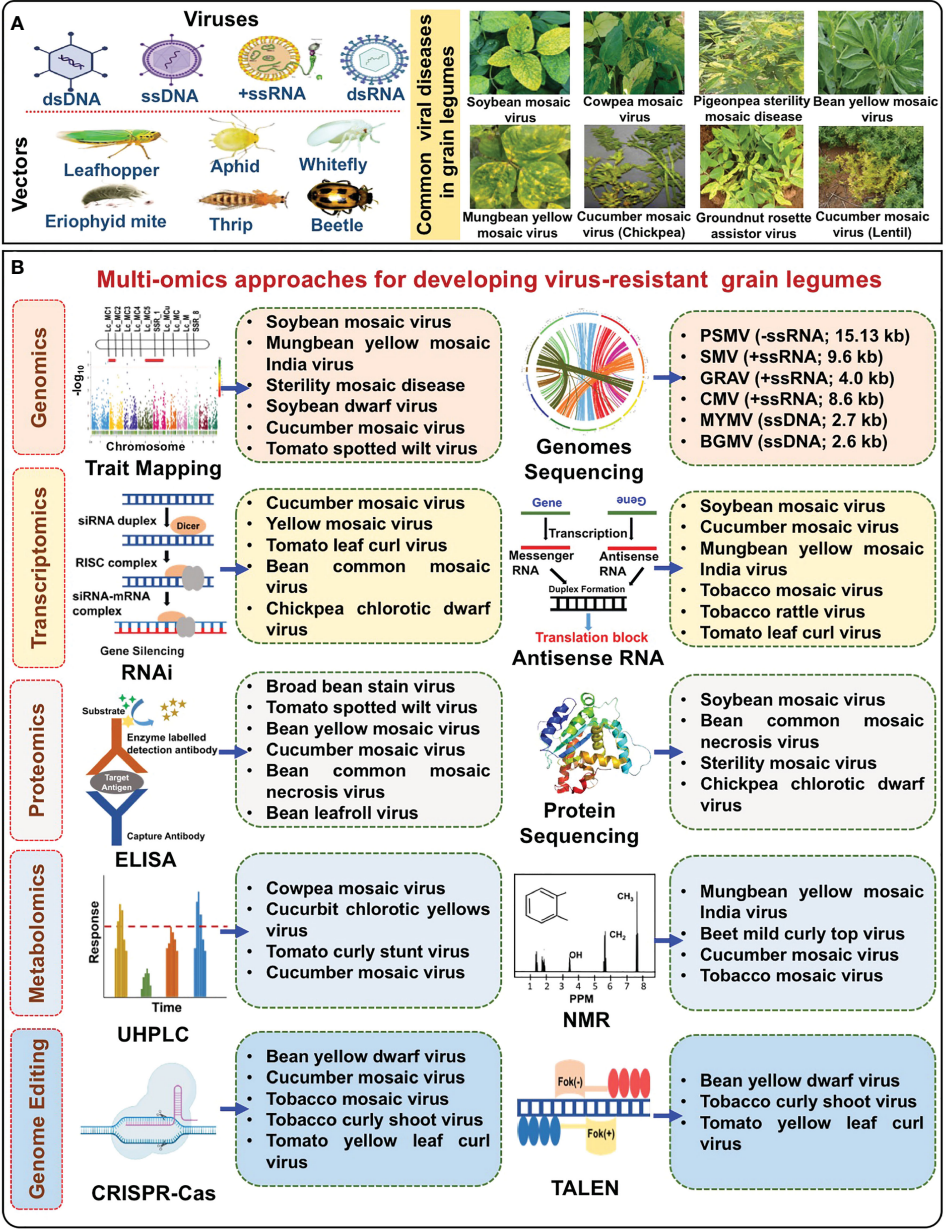
Multi-omics approaches for developing virus-resistant grain legumes. **(A)** Vectors like leaf hoppers, aphids, thrips, whiteflies, and eriophyid mites transmit five kinds of viruses, dsDNA, ssDNA, –ssRNA, +ssRNA, and dsRNA in grain legumes causing viral diseases such as soybean mosaic virus, cowpea mosaic virus, sterility mosaic disease, and rosette. **(B)** Approaches like genomics, transcriptomics, proteomics, metabolomics, and genome editing are used in different crops to develop resistance to viral diseases. Successful examples for each approach are in the boxes. Footnotes: ssDNA, Single-stranded DNA; ssRNA, Single-stranded RNA; PSMV, Pigeonpea sterility mosaic virus; SMV, Soybean mosaic virus; GRAV, Groundnut rosette assistor virus; CMV, Cucumber mosaic virus; MYMV, Mungbean yellow mosaic virus; BGMV, Bean golden mosaic virus; RNAi, RNA interference; ELISA, Enzyme-linked immunosorbent assay; UHPLC, Ultra-high performance liquid chromatography; NMR, Nuclear magnetic resonance spectroscopy; CRISPR-Cas, Clustered regularly interspaced short palindromic repeats-Cas; TALEN, Transcription activator-like effector nucleases.

Biparental QTL mapping is an important molecular approach for improving our understanding of the genetic control of viral disease resistance in grain legumes. Several QTL contributing to various virus diseases in grain legumes have been identified (Blair et al., 2007; Zheng et al., 2014; Ma et al., 2016; Mathivathana et al., 2019) (**Figure 1**). The previous section mentioned the resistance gene(s) controlling SMV; subsequent biparental QTL mapping helped discover the QTL controlling resistance against various strains of SMV (Wang et al., 2011; Zheng et al., 2014; Ma et al., 2016; Karthikeyan et al., 2017; Rui et al., 2017; Liu et al., 2019). Wang et al. (2011) reported that a single dominant gene on chromosome 2 flanked by BARCSOYSSR_02_0610 and BARCSOYSSR_02_0616 governed the genetics of SMV (SC18) resistance in a Kefeng No.1 × Nannong 1138-2 population. Further, QRT-PCR analysis identified five candidate genes. A single dominant allele *Rsv1-h* controlled resistance against multiple SMV strains in soybean cultivar Suweon 97 (Ma et al., 2016), but its chromosomal position was not detected. Subsequently, Zheng et al. (2014) revealed that a single dominant gene *R_SC3Q_* existing on chromosome 13 flanked by BARCSOYSSR_13_1114 and BARCSOYSSR_13_1136 with five underlying candidate genes *Glyma13g25730*, *25750*, *25950*, *25970*, and *26000* controlled the genetics of resistance of SMV SC3 based on F_1_, F_2_, and F_2:3_ populations developed from Qihuang 1× Nannong 1138-2. Likewise, Ma et al. (2016) reported that a single dominant gene controlled the genetics of SMV SC6-N and SC7-N strains from Suweon 97 × Williams 82. Further SSR marker analysis in the F_2_ population mapped *Rsv1-h* to 97.5 kb in the *Rsv1* locus on chromosome 13, with eight possible candidate genes disclosed in this genomic region. Two important genes (*Glyma13g184800* and *Glyma13g184900*) encoding CC-NBS-LRR proteins were identified as candidate genes for *Rsv1-h* (Ma et al., 2016). Similarly, a novel locus *Rsc15* conferring SMV (SC15) was mapped to a 95 kb genomic region on chromosome 6 flanked by SSR_06_17 and BARCSOYSSR_06_0835 markers with candidate genes *Glyma.06g182600*, *Glyma.06g175100* and *Glyma.06g184400* encoding receptor-like kinase, and *Glyma.06g182900* and *Glyma.06g183500* encoding serine/threonine kinase (Rui et al., 2017). For SMV (strain SC5) resistance, a combined approach of Mendelian genetics, biparental QTL mapping, fine mapping, and functional genomics was used (Karthikeyan et al., 2017). Mendelian genetics indicated that the disease resistance was dominant and controlled by a single dominant gene. Phenotyping and high-density linkage mapping in 427 recombinant lines positioned the resistant genomic region on chromosome 2 within a 500 kb interval with 11 putative candidate gene(s) (Karthikeyan et al., 2017). Functional validation of these candidate genes indicated *Glyma02g13495* as the most resistant gene against the SC5 strain (Karthikeyan et al., 2017). Likewise, Karthikeyan et al. (2018) conducted combined classical genetics and biparental QTL mapping to unearth the genetic determinant controlling SMV (strain SC20) resistance. The classical genetics-based study indicated that a single dominant gene governed SMV SC20 resistance. Linkage mapping analysis identified the resistance genomic region on chromosome 13 flanked by BARCSOYSSR_13_1099 and BARCSOYSSR_13_1185 markers (see **Table 3**). The genomic region was narrowed to 79 kb with seven potential candidate genes, of which *Glyma.13G194700* and *Glyma.13G195100*, encoding Toll Interleukin Receptor-nucleotide-binding-leucine-rich repeat resistance proteins, were the most likely candidate genes contributing to resistance. On chromosome 13, a QTL *Rsc18* controlling resistance against SMV strain SC18 was mapped to a 415.357 kb region with three underlying candidate genes: one NBS-LRR type gene and two serine/threonine protein type genes (Liu et al., 2022a).

**Table 3:** List of QTL/gene(s) conferring resistance against various viral diseases in grain legumes.

Crop	Disease	Mapping population	QTL name	Marker used	Linkage map	Phenotypic variation (%)	References
Common bean	Bean common mosaic virus	–	Gene *bc-u*, *bc-1*, *bc-1^2^*, *bc-2*, *bc-2^2^*, and *bc-3*	–	–	–	Feng et al. (2018)
		DOR364 × G19833	*bc-1*	SCAR	–	–	Blair et al. (2007)
		–	*bc-u, bc-1, bc-2*, and *bc-3*	–	–	–	Feng et al. (2018)
	Bean yellow mosaic virus	–	*By-1* and *By-2*	–	–	–	Hart and Griffiths (2015); Schroeder and Provvidenti (1968)
	Bean dwarf mosaic virus	Moncayo × Primo, RIL	*Bct*,	–	–	–	Miklas et al. (2009)
	Bean golden yellow mosaic virus	PR9556-158 × PR9556-171, F_1_, F_2_, F_2:3_, F_3:4_, and BCs	*Bgp-1* (dominant gene)	–	–	–	Román et al. (2004)
		DOR364 × G19833 and BAT93 × Jalo EEP558	*bgm-1*	SCAR	Chr 05		Blair et al. (2007)
		DOR364 × XAN 176, RIL DOR 476 × SEL 1309, RIL	*BGY4.1*, *BGY7.1*, and *BGY8.1*; *bgm-1* candidate gene	SNP	Chr 03	–	Soler-Garzón et al. (2021)
Cowpea	Cowpea severe mosaic virus	F_1_, F_2_, BC_1_, BC_2_	Three genes	–	–	–	Umaharan et al. (1997a)
Groundnut	Tomato spotted wilt virus	Tifrunner × GT-C20, RIL	11 QTL	SSR	A04, A01A09, B02, B04, B10	7–14	Pandey et al. (2017)
		SunOleic 97R × NC94022, RIL(140)	3 QTL	SNP	A01	37%	Agarwal et al. (2019)
Mung bean	Mung bean yellow mosaic virus	*Vigna radiata* × *V. umbellata* interspecific and RILs	*qMYMV4-1*	SNP	LG4	10–20	Mathivathana et al. (2019)
		*Vigna radiata* NM92 × *V. radiata* ssp. *sublobata* TC1966, RILs	Three major and three minor QTL	RAPD, SCAR, CAPs and SSR	LG9, LG8 and LG7	22–59	Chen et al. (2013)
		NM10-12-1 × KPS2, RIL	*qYMIV1, qYMIV2, qYMIV4* and *qYMIV5*	SSR	–	6.2–22	Kitsanachandee et al. (2013)
Pigeon pea	Pigeon pea sterility mosaic	ICPL 20096 × ICPL 332 (PRIL_B), ICPL 20097 × ICP 8863 (PRIL_C) and F_2_ (ICP 8863 × ICPL 87119)	*qSMD11.1, qSMD10.1, qSMD3.1, qSMD7.1, qSMD11.2, qSMD11.3, qSMD11.4, qSMD2.1, qSMD2.2, qSMD2.3*, and *qSMD10.1*	SNP	LG2, LG3, LG7, LG10, and LG11	5.2–34.3	Saxena et al. (2017)
		BSMR 736 × ICP8863	*SV1* and *SV2*	–	–	–	Daspute et al. (2014)
		ICP 8863 × ICPL 20097, TTB 7 × ICP 7035, F_2_	Six QTL including *qSMD4*	SSR	LG7	24.7	Gnanesh et al. (2011)
		ICP 7035 × ICP 8863 and ICP 7349 × ICP 8863	Single gene with three alleles	–	–	–	Srinivas et al. (1997a)
		ICP 7035 × BDN1 and ICP 7349 × BDN1, ICP7349 × LRG30 and ICP8850 × LRG30	Two genes	–	–	–	Srinivas et al. (1997b)
		ICPL 20096 × ICPL 332, RIL	*C. cajan_01839, C. cajan_07067, C. cajan_15535*, and *C. cajan_01839*	SNP	LG2, LG8, and LG11	–	Singh et al. (2016)
Soybean	Soybean mosaic virus	–	*Rsv1*	–	Chr 13		Hayes et al. (2004)
		–	*Rsv3*		Chr 14		Suh et al. (2011)
		–	*Rsv4*		Chr 02		Gunduz et al. (2004)
		–	*Rsv5*	–	Chr 13		Zheng et al. (2005)
		D26 × L68, F_2:3_; V94-5152 × Lee68, RIL	*Rsv4*	SSR	Chr 02	–	Maroof et al. (2010)
		JD12 × HT, RIL	*qTsmv-13, qTsmv-2*, and *qTsmv-3, Glyma.03G00550* and *Glyma.03G00570*	SNP	–	–	Lin et al. (2020)
		Haman × Ilpumgeomjeong, Backcross	*Rsv4*	SNP	–		Ilut et al. (2016)
		Suweon 97 × Williams 82, F_2_	*Rsv1-h locus, Glyma13g184800*, and *Glyma13g184900*	SSR	Chr 13		Ma et al. (2016)
		Raiden × Williams 82, F_2_	*Glyma.13g184800* and *Glyma.13g184900*	SSR and SNP	Chr 13		Wu et al. (2019)
		_	*Rsc15, Glyma.06g175100* and *Glyma.06g184400, Glyma.06g182900* and *Glyma.06g183500*	SSR	Chr 06	–	Rui et al. (2017)
		Kefeng No.1 × Nannong 1138-2, F_2_	*Rsc1-DR*	SSR	Chr 02		Jin et al. (2022)
		Kefeng No.1 (resistant) × Nannong 1138-2	15 genes	SNP	Chr 02		Yan et al. (2015)
		Kefeng No.1 × Nannong 1138-2, F_2_, F_2:3_, and RIL	*Glyma02g13310, 13320, 13400, 13460*, and *13470*	SSR	Chr 02		Wang et al. (2011)
		Qihuang-1 × NN1138-2, F_1_, F_2_ and RIL	*Glyma.13G194700* and *Glyma.13G195100*	SSR	Chr 13		Karthikeyan et al. (2018)
		Kefeng-1 × NN1138-2, F_1_, F_2_, RIL	*Glyma02g13495*	SNP			Karthikeyan et al. (2017)
	Tobacco ringspot virus	GWAS	Two candidate *LRR-RLK* genes	SNP	Chr 02	–	Chang et al. (2016)
	Bean common mosaic virus	Raiden × Williams 82, F_2_	*Rsv1*	SSR and SNP	–		Wu et al. (2019)
Urdbean	Mung bean yellow mosaic virus	MDU 1 × TU 68	*qMYMVD*_*60*		LG10	21	Subramaniyan et al. (2022)
		Pant U-84 × UL-2, F_2_, BC, UPU-2 × UL-2, F_2_, BC	Two recessive genes	–	–	–	Singh (1980)
		T-9 (YMV-tolerant) × T-9 (YMV-susceptible), F_1_, F_2_, F_3_	Monogenic	_	–	–	Basak et al. (2005)
		–	*YR4* and *CYR1*	SSR	–		Maiti et al. (2011)
		KUG253 × Mash114, RIL	*qMYMIV6.1.1*	KSAP	Chr 06	70	Dhaliwal et al. (2022)

Maroof et al. (2010) assessed two RIL-based mapping populations (D26 × Lee68 and V94-5152 × Lee68) to understand the molecular genetics of the *Rsv 4* gene conferring SMV resistance. The soybean populations were genotyped with newly developed SSR and SNP markers from whole-genome shotgun sequencing, shortening the genetic interval containing *Rsv4* to 0.7 cM and 1.3 cM in the V94-5152 × Lee68 and D26 × Lee68 mapping populations, respectively. The underlying candidate gene(s) encoded AGAMOUS-LIKE 28 TF and myb-like protein (Maroof et al., 2010). Subsequently, Ilut et al. (2016) fine-mapped this gene to a ~120 kb interval using a BC_3_F_2_ backcross population developed from Haman × Ilpumgeomjeong (see **Table 3**). Furthermore, haplotype analysis using SNP markers resolved the association signal to a ~94 kb region that contained two *Rsv4* haplotypes. This *Rsv4* locus was cloned *via* positional cloning, encoding an RNase H-family protein with dsRNA degradation (Ishibashi et al., 2019). Fine mapping of the genomic region within 186 kb flanked by SSR markers BS020610 and BS020620 on chromosome 2—conferring resistance against the SMV strain SC1 in the F_2_ population containing 218 individuals—identified 14 genes (Jin et al., 2022), of which the *Rsv4* allele (designated *Rsc1-DR*) was accountable for resistance to SMV-SC1 (Jin et al., 2022). Likewise, cloning and functional analysis of MADS-box transcription factor *GmCAL* from soybean-resistant Kefeng-1 cultivar showed that overexpression of this gene conferred resistance against SMV-SC3, SMV-SC7, and SMV-SC8 in SMV-susceptible NN1138-2 soybean cultivar (Ren et al., 2022).

Lin et al. (2020) conducted genetic analysis in JD12 × HT F_2_ and recombinant inbred lines population to identify the QTL/genomic region controlling resistance against the novel recombinant SMV strain. They found that the resistant gene was dominant and governed by a single gene, with one QTL for resistance (*qTsmv-13*) and two QTL for tolerance (*qTsmv-2* and *qTsmv-3*) against the novel recombinant SMV strain (see **Table 3**). Comparative analysis of known resistance genes indicated that *qTsmv-13* and *qTsmv-2* corresponded to *Rsv1* and *Rsv4*, respectively. Map-based cloning of *qTsmv-3* was delimited to 86 kb. Of the five identified candidate genes underlying the genomic region, *Glyma.03G00550* (multidrug and toxic compound extrusion transporter gene) was a potential candidate gene for resistance against the disease. For combined resistance against SMV and BCMV in soybean, Wu et al. (2019) investigated the genetics of the gene controlling resistance against both diseases, reporting that a single dominant gene controlled each disease. Bulk segregation analysis indicated that the BCMV-resistance gene was linked to the SMV-resistant *Rsv1* complex locus, with the SMV-resistant gene *Rsv1-r* flanked by BARCSOYSSR_13_1075 and BARCSOYSSR_13_1161 markers, and the BCMV-resistance gene flanked by BARCSOYSSR_13_1084 and BARCSOYSSR_13_1115 markers (Wu et al., 2019). Further, the authors narrowed the SMV- and BCMV-resistance genes to ~154.5 kb between two SNP markers (SNP-38 and SNP-50).

MYMIV is an important yield constraint in mungbean. To elucidate the QTL controlling MYMIV resistance in mungbean, an NM92 (MYMIV-tolerant cultivated line) × TC1966 RIL population was screened for MYMIV resistance under field conditions, with the population genotyped using RAPD, AFLP, SCAR, and CAP markers (Chen et al., 2012). Three QTL on LG9 (MYMIVr 9_6.4, MYMIVr 12.7, and MYMIVr 9_25) and MYMIVr 8_29.1 on LG8 contributed to MYMIV resistance (Chen et al., 2012) (see **Table 3**). MYMV is a major viral disease causing serious yield limitations in urdbean. Five QTL, including one major QTL *qMYMV4-1* on chromosome 4, were identified in an interspecific cross *Vigna radiata* × *V. umbellata* using the genotyping-by-sequencing method and phenotyping the population under field conditions for two consecutive years (Mathivathana et al., 2019). Further, Subramaniyan et al. (2022) conducted phenotyping and QTL analysis of mapping population developed from MDU1× TU68 cross to uncover the genetic determinant/genomic region controlling MYMV resistance. Classical genetic analysis indicated inhibitory gene action with two genes controlling MYMV resistance, while QTL analysis suggested one major QTL *qMYMVD*_*60* flanked by CEDG180 and CEDG116 marker at LG 10 controlling MYMV resistance (see **Table 3**). Recently, to introduce MYMIV resistance from *Vigna umbellulata* to urdbean, Dhaliwal et al. (2022) used a QTL-seq-based approach to identify *qMYMIV6.1.1*, a major QTL spanning 3.4 Mb on chromosome 6, contributing 70% phenotypic variation. Further, the authors elucidated three possible candidate genes (*serine threonine kinase*, *UBE2D2*, and *BAK1/BRI1-ASSOCIATED RECEPTOR KINASE*) underlying the identified genomic region.

BGYMV is an important disease causing significant yield losses in common bean. A SCAR marker SR2, linked to a *bgm-1* resistance gene, was developed and mapped 7.8 cM from the resistance gene in a DOR476 × SEL1309 RIL population (Blair et al., 2007). The SR2 marker was located at the end of chromosome 5 in DOR364 × G19833 and BAT93 × Jalo EEP558 mapping populations. Notably, the *bgm-1* resistance gene was closely related to the *bc-1* resistance gene for BCMV (Blair et al., 2007). Thus, these genomic regions could be targeted for developing combined resistance against BGYMV and BCMV.

## Genome-wide association study capturing viral-disease-resistant genomic regions across the whole genome

The GWAS approach using marker-trait associations and various statistical models could identify the underlying candidate gene(s)/QTL/genomic regions controlling viral disease resistance in various legumes, overcoming the limitations of biparental QTL mapping (Huang and Han, 2014) (**Figure 1**). For example, a comprehensive GWAS for TRSV in a set of 19,652 soybean genotypes using the SoySNP50K iSelect BeadChip detected a single locus associated with TRSV sensitivity on chromosome 2 and predicted two leucine-rich repeat receptor-like kinase genes *Glyma02g13460* and *Glyma02g13470* underlying the locus (Chang et al., 2016). Similarly, Liu et al. (2019) conducted GWAS in two soybean populations containing 409 and 199 genotypes in a SoySNP50K assay to unearth significant marker-trait associations for SMV seed transmission rate, seed coat mottling, and seed yield loss due to SMV infection. The study identified a single locus contributing to SMV seed transmission rate on chromosome 9, loci for seed coat mottling on chromosome 3, and a single locus for seed yield loss due to SMV infection on chromosome 3 (Liu et al., 2019). An earlier GWAS investigating SMV(SC7) resistance in 165 soybean genotypes identified three genes, homologous to *WRKY72, eEF1Bβ*, and *RLP9*, conferring disease resistance in *Arabidopsis* on chromosomes 1, 3, and 9, respectively (Che et al., 2017). Combined recombinant inbred line based linkage and GWAS analysis allowed deciphering a major QTL *qSMV13* on chromosome 13 conferring resistance against SMV (SC3 and SC7) strain explaining 71% and 76% PV, respectively (Chu et al., 2021).

A GWAS of 182 common bean genotypes belonging to the Durango diversity panel in association with 1.26 million SNPs detected significant marker-trait associations for resistance against BCMNV isolates NL-8 and NL-3 on PV03 and PV05 chromosomes that corresponded to *bc-1* and *bc-u* resistance gene loci, respectively, elucidating two candidate genes for *bc-1* (*Phvul.003G038700* and *Phvul.003G038800*) and one bZIP protein gene for *bc-u* (*Phvul.005G124100*) (Soler-Garzón et al., 2021). Further advances in GWAS models could help minimize population-structure-related problems.

## Progress of functional genomics: discovery of candidate resistance gene(s) with putative functions

Functional genomics is a powerful approach for discovering candidate gene(s) related to various disease resistance with putative functions, including viral diseases in grain legumes. Recent advances in RNA-seq-based transcriptome sequencing have offered novel insights into the molecular mechanisms of disease resistance and identified possible candidate gene(s) conferring resistance against various viral diseases in grain legumes (Martin et al., 2016; Dasgupta et al., 2021).

Yuan et al. (2020) used transcriptomic analysis of R and S isogenic lines developed from Qihuang-1 Nannong 1138-2 × Soybean cv. Qihuang-1 subjected to SMV infection at 6, 20, and 48 h post-inoculation (hpi) to elucidate the underlying candidate gene(s) conferring SMV resistance. A further DEGs analysis revealed the downregulation of *Glyma03g28650, Glyma19g31395*, and *Glyma11g33790* encoding calmodulin-like protein in S lines and upregulation of jasmonic acid repressor genes (*TIFY/JAZ*) and abscisic-acid-induced genes (*PP2C3a*) in R lines in response to SMV infection. Likewise, Chen et al. (2022) deciphered the role of genes related to phytohormone-mediated SMV resistance using RNA-seq-based transcriptome analysis in contrasting soybean genotypes, Kefeng-1 (resistant) and NN1138-2 (susceptible). Using DEGs and gene ontology analyses and the functional validation of candidate genes revealed the downregulation of *Glyma.11G239000* and *Glyma.18G018400*, belonging to ethylene-insensitive 3/ethylene-insensitive3-like (EIN3/EIL) protein family, in NN1138-2. At 48 hpi, jasmonic acid repressor genes (*TIFY*/*JAZ*) and *NPR1* involved in the salicylic acid signaling were downregulated in NN1138-2 but upregulated in Kefeng-1 (Chen et al., 2022).

Sun et al. (2022) investigated the role of abundant H_2_O_2_ production in regulating callose deposition on plasmodesmata, mediating the blockage of intercellular transport of SMV infection in soybean. Two genes regulated by H_2_O_2_ (*GmSEOB* and *GmPAP27*) conferred resistance against SMV by positively regulating callose accumulation in response to SMV infection at the transcriptomic and VIGS levels.

Dasgupta et al. (2021) performed RNA-seq-based transcriptome analysis of PMR-1 (resistant) and Pusa Vishal (susceptible) mungbean genotypes, elucidating the participatory role of WRKY, NAC, and MYB transcription factors in mediating YMV resistance. A DEGs analysis revealed that PMR-1 upregulated peroxidase, (S)-2-hydroxy-acid oxidase, and classes of lipoxygenase and downregulated oxidoreductase, 2OG-Fe(II) oxygenase family protein, 4-coumarate:CoA ligase like, and O-methyltransferase encoding genes in response to YMV. Further, the authors validated 11 defense-related transcripts contributing to YMV resistance. In another study, RNA-seq of mungbean elucidated the role of various DEGs related to TFs, hormone signaling, receptor-like kinases, serine/threonine protein kinases, defense, and pathogenesis in mediating MYMV resistance (Sudha et al., 2022) (**Figure 1**). In addition, qRT-PCR analysis was used to functionally validate select candidate DEGs related to MYMV defense mechanisms (*Vradi08g04110, Vradi09g06830, Vradi04g07450, Vradi06g13520, Vradi06g11500*, and *Vradi01g04820*).

A PCR-based suppression subtractive hybridization technique identified 345 candidate genes differentially expressed in response to MYMIV infection in urdbean, contributing various cellular functions mediating resistance against MYMIV, including Ca^2+^-mediated signaling, reactive oxygen species generation, phenylpropanoids, and ubiquitin-proteasomal pathways (Kundu et al., 2015). Further, Kundu et al. (2019) performed transcriptome analysis of two contrasting urdbean genotypes, VM84 (resistant) and T9 (susceptible), to decipher the gene(s) and molecular mechanisms mediating MYMV resistance. They discovered 2,158 and 1,679 DEGs from VM84 and T9 in response to MYMV infection, of which NB-LRR, WRKY33, ankyrin, argonaute, and NAC TFs exhibited upregulatory responses in MYMV-resistant VM84, indicating their role in conferring disease resistance.

A transcriptome analysis of a BCMV-susceptible common cultivar (Stringless green refugee) in response to two BCMV infection isolates revealed upregulation of genes related to receptor-like protein kinase, pathogenesis-related proteins, and oxidative-stress-related genes and downregulation of genes related to photosynthetic machinery (Martin et al., 2016). Among the various TF genes, NAC and WRKY_Zn families had increased expression, and Myb_related and bHLH families had reduced expression in response to BCMV infection (Martin et al., 2016), improving our understanding of various gene networks that mediate resistance against BCMV in common bean.

Evidence of non-coding RNAs, including miRNAs, siRNAs, and long non-coding regulating disease resistance, is well established in plants (Jha et al., 2021; Song et al., 2021). Patwa et al. (2021) uncovered 422 differentially expressed miRNAs responding to MYMIV infection in *P. vulgaris.* Validation of selected candidate miRNAs revealed their role in regulating various TFs involved in MYMIV resistance. Illustrations of non-coding RNAs conferring viral disease resistance are limited in grain legumes, requiring further studies on their molecular mechanisms and corresponding target gene(s) conferring viral disease resistance.

## Genetic engineering approach for designing virus-disease-resistant grain legumes

**Figure 2:**
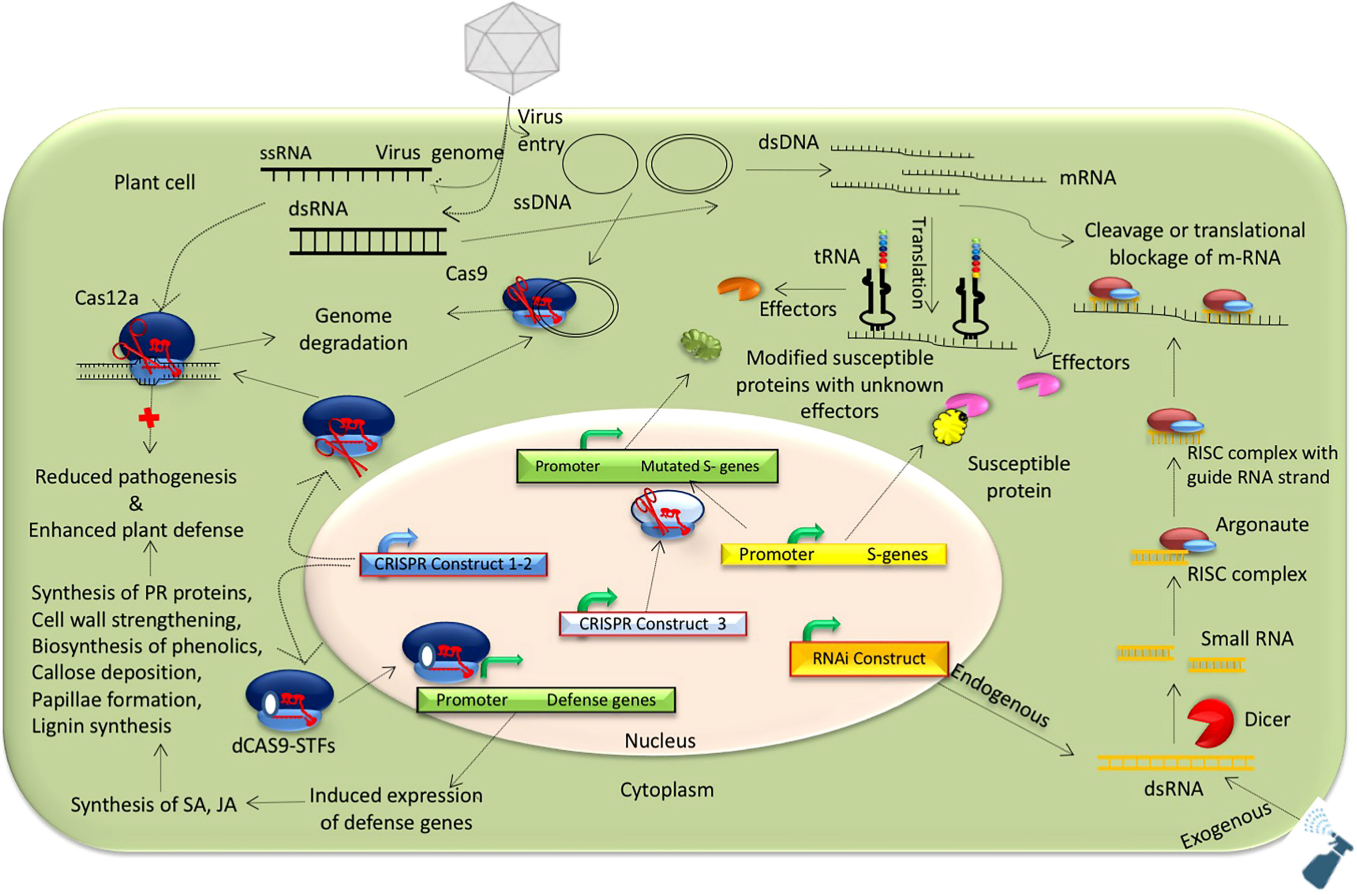
Possible mechanisms for developing virus-resistant grain legumes using RNAi and genome editing technologies.

Genetic engineering is a powerful option for developing virus-resistant grain legumes (Bonfim et al., 2007; Zhang et al., 2012; Aragão et al., 2013; de Paula et al., 2015) (**Figure 1**), especially when there are limited options for host plant resistance. Different functional genomic approaches spanning transgenic to non-transgenic technologies can be used to design virus-resistant plants. Transgenic technologies are the primary initiative for plant virus management, with success stories in papaya and cucurbits (Kreuze and Valkonen, 2017) but limited application in grain legumes. There are only a few examples of RNAi-mediated transgenic legumes developed for virus resistance, mainly in cowpea, common bean, and soybean, with pathogen-derived resistance (expressing the virus gene/genome) adopted. An RNAi strategy was used to manage cowpea severe mosaic virus and CABMV in transgenic cowpea expressing proteinase cofactor and coat protein for the respective viruses (Cruz and Aragão, 2014). Similarly, enhanced resistance in cowpea against multiple Begomovirus infections was achieved through the expression of AC2 and AC4 and fusion of AC2 and AC4 gene-based hairpin constructs (Kumar et al., 2017). Further, an RNAi strategy was used in soybean to confer resistance against multiple potyviruses. The first SMV-resistant transgenic soybean lines were developed by integrating an inverted repeat sequence of HC-Pro of SMV (Gao et al., 2015). Likewise, introducing a 248 bp inverted repeat of the replicase (nuclear inclusion b, Nib) gene from the SMV SC3 strain into soybean resulted in transgenic soybean plants with stable resistance against five strains of SMV (SC3, SC7, SC15, SC18, and a recombinant SMV), BCMV, and watermelon mosaic virus (WMV) (Yang et al., 2017). Inserting a 302 bp inverted repeat of the P3 cistron, isolated from SMV strain SC3 resulted in RNAi-mediated genetically engineered soybean lines with stable resistance against multiple virus strains of SMV, BCMV, and WMV (Yang et al., 2018). RNAi strategies have also been used to combat bean golden mosaic virus (BGMV) in common bean by silencing the rep (AC1) viral gene (Bonfim et al., 2007; Aragão et al., 2013; de Paula et al., 2015) and to prevent whitefly (*Bamicia tabaci*) infection by suppressing the ATPase (Bt-vATPase) gene in *B. tabaci* (Ferreira et al., 2022). Thus, pathogen-derived transgenic resistance can be achieved using an RNAi strategy to develop virus resistance in legumes (see **Figure 2**).

The host-derived strategy (targeting host-susceptible genes) is another option for transgenic resistance against plant viruses in grain legumes. This strategy can be used to silence the host eukaryotic translation initiation factor to prevent pathogen replication or overexpress resistance gene(s) and transcription factors (Chattopadhyay et al., 2019). The silencing of eukaryotic translation initiation factor 4E (eIF4E) in soybean *via* RNAi technology resulted in broad-spectrum resistance against multiple potyviruses, including SMV strains (SC3/7/15/18 and SMV-R), BCMV, and WMV (Gao et al., 2020). In contrast, overexpression of GmKR3 (TIR-NBS-LRR type R gene) in transgenic soybean showed high resistance against SMV, BCMV, WMV, and secovirus BPMV by mediating ABA signaling (Xun et al., 2019) (**Table 4**). Similarly, the overexpression of GmAKT2 in transgenic soybean significantly increased K^+^ concentrations and enhanced SMV resistance (Zhou et al., 2014). Further, metabolic engineering of susceptible soybean lines enhanced SMV resistance by overexpressing a cinnamyl alcohol dehydrogenase-coding *GsCAD1* gene (Xun et al., 2022) and purple acid phosphatase encoding *GmPAP2.1* gene (Widyasari et al., 2022).

**Table 4:** List of transgenes conferring resistance against various viral diseases in grain legumes.

Crop	Disease	Gene	Function	Method used	Reference
Common bean	Bean golden mosaic virus	*AC1*	Silencing of *AC1* gene rendered resistant against the infecting virus	RNAi-mediated	Bonfim et al. (2007); Aragão et al. (2013)
		*Rep gene*	*–*	RNAi-mediated	de Paula et al. (2015)
Cowpea	Mungbean yellow mosaic India virus	*AC2*, *AC4*	Conferred resistance against mungbean yellow mosaic India virus	RNAi-mediated	Kumar et al. (2017)
Soybean	Soybean mosaic virus	*SMV- HC - Pro* genes	Resistant to soybean mosaic virus	RNAi-mediated	Gao et al. (2015)
		*Replicase*	Resistant to soybean mosaic virus	RNAi-mediated	Yang et al. (2017)
		*AGO1, AGO2, DCL1* and *DCL2*, *and NBS-LRR*	miR1507a, miR1507c, miR482a, miR168a, miR1515a control the expression of *AGO1, DCL1, DCL2*, and five *NBS-LRR* genes	RNAi-mediated	Bao et al. (2018)
		*Rsv1*	Silencing of *GmEDR1, GmEDS1, GmJAR1, GmHSP90*, and *GmPAD4* caused loss of extreme resistance of *Rsv1* gene	Virus-induced gene silencing	Zhang et al. (2012)
	Soybean mosaic virus, bean common mosaic virus, watermelon mosaic virus and bean pod mottle virus	SMV P3 cistron, Ribonuclease gene PAC1, factor 4E and GmKR3	Resistant to all these viral diseases	RNAi-mediated and Agrobacterium-mediated	Yang et al. (Yang et al., 2018; Yang et al., 2019); Xun et al. (2019); Gao et al. (2020)

Non-transgenic approaches have become popular as they avoid time constraints and overcome transgenics-associated risks (Teli et al., 2020). Exogenous application of RNAi-inducing double-stranded RNA (dsRNA) molecules is an alternative approach to generating antiviral RNAi in plants (Mitter et al., 2017), especially when genetic transformation and regeneration are time-consuming. The successful delivery of dsRNA within host cells is crucial for executing RNAi-mediated plant defenses against viruses. In cowpea, the bioefficacy of dsRNAs targeting the potyviral nuclear inclusion b (NIb) protein and coat protein-encoding gene of BCMV was reported (Worrall et al., 2019). Similarly, direct foliar application of dsRNA derived from the full-length NSs gene of groundnut bud necrosis virus significantly reduced the virus infection in cowpea (Gupta et al., 2021). However, dsRNA delivery is an issue, particularly for field applications. Delivering dsRNA through layered double hydroxide nanoparticles prevented virus replication better than naked dsRNA as it facilitated the slow release of dsRNAs and long-term viral protection (Mitter et al., 2017). Furthermore, targeting vector transmission through exogenous dsRNA application could be an innovative option for developing a tailor-made bio-pesticide. In cowpea, bio-clay mediated application of dsRNA prevented aphid-mediated transmission of BCMV (Worrall et al., 2019). However, these strategies require testing under field conditions to predict possible environmental risks.

Likewise, genome editing has emerged as a versatile tool for designing viral-disease-resistant crop plants, including grain legumes (Langner et al., 2018), and overcoming public and scientific concerns related to the development end release of transgenic plants. Genome editing technologies have been used for genome engineering in many crop plants, but there is limited evidence in grain legumes (Gayen and Karmakar, 2021), except for cowpea (Ji et al., 2019; Juranić et al., 2020; Che et al., 2021), chickpea (Badhan et al., 2021), and soybean (Al Amin et al., 2019; Bao et al., 2019; Bao et al., 2020), due to hurdles related to *in vitro* gene transfer and poor regeneration (Bhowmik et al., 2021). Despite that, gene-editing technologies have opened up new opportunities for multiple trait improvement in grain legumes, especially for plant virus resistance. So far, CRISPR-Cas9-mediated genome editing has been used to manage different virus pathogens, including Merremia mosaic virus (Ali et al., 2016), tobacco rattle virus (Ali et al., 2015), beet necrotic yellow vein virus, pea early browning virus (Ali et al., 2018), tobacco mosaic virus (Cody et al., 2017), beet yellow dwarf virus (Liang et al., 2016), and TYLCV (Tashkandi et al., 2018). However, notable examples are only available for soybean and mungbean. CRISPR/Cas9-mediated multiplex gene-editing technology was used to increase isoflavone content for enhancing SMV resistance in soybean by editing two flavanone‐3‐hydroxylase genes (*GmF3H1*, *GmF3H2*) and one flavone synthase (*GmFNSII-1*) gene (Zhang et al., 2020). Thus, CRISPR/Cas-mediated metabolic engineering could be an option for virus resistance in legumes. Further, manipulation of host-susceptible factors, viz., eukaryotic translation initiation factor 4E (eIF4E), could help achieve potyvirus resistance in different legume crops. CRISPR/Cas9-mediated editing of virus genomes can also be used to protect plants from virus infection (see **Figure 2**). The CRISPR/Cas9 system with two guide RNA cassettes was used to edit the MYMV genome in mung bean, targeting the replicase enzyme (AC1) and coat protein (AV1) encoding genes (Talakayala et al., 2022). Such multiplex strategies assembling different gRNA cassettes for several viral genomes could be suitable for managing multiple plant viruses simultaneously.

## Emerging breeding tools to design grain legumes with viral disease resistance

Genomics selection facilitates the selection of genotypes with high genetic merit by calculating the genomic-estimated breeding value of individuals based on various prediction models using genotypic and phenotypic information from the ‘training population’ (Meuwissen et al., 2001; Lorenz et al., 2011). Thus, this breeding tool can be used to select superior progenies or genotypes from large germplasm sets without needing phenotypic evaluation. For example, a genomic prediction model incorporating GAPIT and rrBLUP was used to predict TRSV sensitivity in soybean, with 140 soybean PIs used as the validation population and 557 soybean PIs used as the training population (Chang et al., 2016). An additional 55 soybean accessions were evaluated for TRSV sensitivity to assess the model’s prediction accuracy. The results revealed a correlation of 0.67 (P < 0.01) between actual and predicted severities of TRSV (Chang et al., 2016). More recently, a smart breeding scheme, ‘integrated genomic–enviromic prediction’, or an advanced form of genomic prediction constituting multi-omics data, big data, and artificial intelligence, was proposed for prediction-based crop redesign (Xu et al., 2022). This high throughput technology could open up avenues for designing viral-disease-resistant grain legumes.

‘Rapid generation advancement’ protocols have been developed in various crop plants, including grain legumes, shortening the breeding cycle and advancing mapping populations (Watson et al., 2018; Hickey et al., 2019). These protocols have optimized photoperiods, daylengths, and temperatures in soybean, cowpea, chickpea, and pigeon pea by growing them in regulated growth chambers (Samineni et al., 2019; Saxena et al., 2019; Fang et al., 2021; Edet and Ishii, 2022). Thus, harnessing the potential of this technique could help develop viral disease resistance in grain legumes.

Advances in next-generation sequencing-based approaches and bioinformatic analyses have facilitated the identification of target genomic regions conferring disease resistance in various crops, including grain legumes (Thudi et al., 2020; Gangurde et al., 2022). Further, these technologies have enabled WGRS of large-scale global germplasm and pangenome construction for various legumes, including soybean (Li et al., 2014), chickpea (Varshney et al., 2021), pigeon pea (Zhao et al., 2020), cowpea (Liang et al., 2022), and mungbean (Liu et al., 2022b). Thus, these genomic resources could help underpin novel presence/absence variations contributing to various viral-disease-resistance genes and pathogenesis genes for developing next-generation viral-disease-resistant grain legumes.

Furthermore, rapid developments in synthetic biology have enabled desirable crop designs. Synthetic biology approaches involve designing genetic circuits, synthetic promoters (SPs), and synthetic transcription factors (STFs) that facilitate the modification of crop plants by remodeling the gene/genome structure, reprogramming gene function, and engineering existing metabolic pathways (Liu and Stewart, 2016). Adequate advances have been made in designing SPs and STFs for regulating specific genes involved in multiple trait improvement in crop plants, including biotic and stress tolerance (Dey et al., 2015; Yasmeen et al., 2023). Synthetic promoters are designed rationally and constructed for specific binding to upstream motifs or native core promoter sites (Khan et al., 2023) so that external stimuli can regulate gene expression (Rushton et al., 2002). This strategy is mostly suitable for enhancing biotic stress tolerance when pathogen-inducible SPs can be employed against diverse pathogens (Khan et al., 2023), as exemplified against *Ascochyta rabiei* (Shokouhifar et al., 2019). Similarly, STFs can be constructed by fusing various DNA binding domains (DBDs) with an activator/repressor domain and nuclear localization signals for regulated gene expression in plants. Nowadays, synthetic DBDs based on C2H2 zinc-finger (ZF) proteins, transcription activator-like effectors (TALEs), and CRISPR/dCas9 can be designed easily for specific binding to promoter sequences of endogenous genes or transgenes (Liu and Stewart, 2016). These STFs, viz., ZF-TFs, TALE-TFs, and dCas9-TFs, can regulate endogenous genes or transgenes to confer defense against broad-spectrum pathogens such as plant viruses. Sometimes, SPs are not adequate for obtaining higher expression levels of transgenes. In such situations, SFs can be delivered with SPs to maximize gene expression at full strength and specificity. Hence, simultaneous expression of SPs with corresponding synthetic TFs is necessary for transgene activation in legumes targeted against plant viruses. Therefore, synthetic biology tools like SPs and STFs can provide tremendous advantages over their natural counterparts for designing legumes resistant to multiple viruses.

Likewise, to overcome transgenics-related issues, CRISPR/Cas9-based genome editing has emerged as a versatile tool for designing viral-disease-resistant crop plants, including grain legumes (Langner et al., 2018). Notable examples of viral diseases include *Merremia mosaic virus* (Ali et al., 2016), tobacco rattle virus (Ali et al., 2015), beet necrotic yellow vein virus, and pea early browning virus (Ali et al., 2018), tobacco mosaic virus (Cody et al., 2017), beet yellow dwarf virus (Liang et al., 2016), and TYLCV (Tashkandi et al., 2018). Similarly, a CRISPR/Cas9-mediated approach was used to manipulate single-stranded DNA-A of AC1 (rep protein) and AV1 (coat protein) for developing MYMV-resistant mungbean (Talakayala et al., 2022). However, the development of viral-resistant grain legumes using genome editing tools remains low. Hence, optimizing the transformation protocol and genome editing tools could help develop grain legumes with virus resistance.

## Integration of “OMICS” technologies with plant breeding for uncovering the virus resistance gene(s)/QTLs and deciphering the complex gene networks of controlling virus resistance in grain legumes

To understand the molecular mechanism of plant - virus interaction, resistance mechanism and complex gene networks controlling virus resistance in plant including grain legume, a multi-layered scientific approach is needed (Fernie and Schauer, 2009; Weckwerth, 2011). Plant ‘omics’ encompassing genomics, transcriptomics, proteomics, metabolomics, epigenomics, and phenomics and genetic engineering including RNAi and synthetic biology in concert with classical and emerging plant breeding approaches could greatly assist in elucidating the candidate gene(s) conferring virus resistance along with their precise function (Fernie and Schauer, 2009; Weckwerth, 2011; Ghatak et al., 2017a, Ghatak et al., 2017b; Ghatak et al., 2018; Weckwerth et al., 2020).

## Limitations of breeding and biotechnological tools for developing virus-resistant legumes

Plant breeding is a practical approach for developing virus-resistant grain legumes. However, transferring disease-resistant gene(s)/QTL is time-consuming, involving the hybridization and selection of disease-resistant plants. Marker-assisted plant breeding can sometimes support the process, but the poor associations between markers and traits make the markers ‘breeder unfriendly,’ which may not transfer well between populations. Biotechnological and molecular biological strategies for developing virus-resistant legumes also face limitations. The scarcity of genomic information for different legumes adds to the difficulty. As grain legumes are recalcitrant to transformation, a precise and robust protocol should be necessary to deliver the genome engineering machinery into germline cells for developing genome-edited plants (Zaidi et al., 2020). Currently, the delivery of CRISPR/Cas cassettes into plant cells occurs through Agrobacterium-mediated transformation, particle bombardment, or protoplast transfection strategy (Zaidi et al., 2020), all of which involve tedious tissue culture procedures. To bypass the complexity of plant tissue culture, plant virus-based vectors can be used for the successful delivery of CRISPR/Cas reagents, such as CRISPR/Cas proteins, guide RNA (gRNA), and ribonucleoproteins (RNP), into plant cells (Zhang et al., 2022). The virus‐inducible genome editing (VIGE) system based on plant virus vectors has been applied successfully for genome editing and antiviral breeding (Ji et al., 2018). VIGE is highly suitable for precise genome editing, minimizing ‘off-target mutations’ common in ‘normal’ genome editing (Hahn and Nekrasov, 2019). Off-target mutations are highly desirable when developing CRISPR/Cas9-based genome editing systems against plant DNA viruses infecting grain legumes.

The design of virus-resistant legumes *via* genome editing targets the virus genome by cleaving through CRISPR/Cas9, which can exert natural selection pressure on the virus population, leading to virus evolution through sequence variation in the virus genome and the emergence of recombinant virus species/strains (especially begomoviruses) resistant to Cas9 cleavage (Cao et al., 2020; Mushtaq et al., 2020). Further, knocking out host susceptibility factors, such as eIF4E, is a common practice for designing potyvirus-resistant plants, but the loss-of-function of the host factor may inhibit plant growth (Gauffier et al., 2016). This problem can be avoided by introducing point mutation (base editing) in the gene to prevent plant virus infection without impairing plant growth (Bastet et al., 2018). Furthermore, the CRISPR-Cas9 edited virus-resistant plant should be rigorously tested in the field under multiple environments to ensure durable resistance against plant viruses (Robertson et al., 2022). A proper regulatory framework should be established before the public release of genome-edited plants. Their classification as ‘GMOs (genetically modified organisms)’ or non-GMOs should also be clarified. Controversy and confusion persist around the GMO and non-GMO classification of genome-edited plants, which could be simply defined based on the presence or absence of foreign DNA. Usually, transgenic expression of the CRISPR-gRNA construct is needed for successful genome editing. However, avoiding transgenic approaches through the transient delivery of CRISPR/Cas and gRNA constructs *via* the VIGE system could make it possible to generate plant genomes free from foreign DNA. Finally, legumes are highly susceptible to infection by multiple viruses, often leading to co-infections. To combat this, a multiplexed CRISPR strategy could be used to design plants resistant to multiple viruses. However, the large construct size required could reduce the efficiency of this strategy. These limitations of biotechnological tools impede the development of virus-resistant legumes, highlighting the need for continued research to overcome these challenges and improve the efficiency of genome-editing techniques.

## Outstanding questions stimulating further research in this area

Several outstanding questions remain regarding the development of virus-resistant legumes. The ever-emerging population of plant viruses poses a major challenge, as virus evolution and the emergence of new virus strains infecting legumes is constant. With the increasing host range of legume-infecting viruses and their co-infections, tackling the problem of emerging virus strains becomes even more difficult in the global warming and climate change scenario. The durability of host resistance is thus always in question. Further, the expression of plant resistance often results in energy loss and yield costs, which should be addressed through advanced breeding and biotechnological approaches.

## Conclusion and future perspective

Global climate change has caused significant challenges for agroecosystems and global food security (Schmidhuber and Tubiello, 2007). The increasing incidence of various diseases, including viral disease, challenges grain legume yields. Moreover, the growing global human population, estimated to reach 9–10 billion by 2030, further pressures global food security (Pérez-Escamilla, 2017). Consequently, food production needs to double to sustainably meet the rising demand for food supply. Harnessing the global genetic diversity of grain legumes with resistance to various viral diseases is a promising and sustainable approach to developing climate-resilient grain legumes with improved viral disease resistance. Pre-breeding approaches and marker-assisted breeding schemes can help transfer gene(s)/QTL conferring resistance against viral diseases to high-yielding grain legumes. Unprecedented advances in sequencing technologies have enabled access to genomic information related to disease resistance and virulence gene(s)/genomic regions across the whole-genome level for designing virus-resistant crop plants. GWAS, WGRS, and pangenome approaches have further underpinned novel resistance R genes and effector encoding genes at the whole-genome level. Furthermore, functional genomics, including transcriptomics, has assisted in the discovery of novel candidate gene(s) conferring disease resistance and the molecular mechanisms of host plant disease resistance. Advances in genetic engineering techniques, such as RNAi technology, can be used to unravel virus-resistance gene function and design virus-resistant grain legume genotypes. However, progress is limited in legumes, which could be accelerated by applying powerful transgene-free, genome-editing technologies targeting viral genomes (e.g., replicase enzyme (AC1) and coat protein (AV1)-encoding genes) and the plant S gene accounting for viral disease development (Zaidi et al., 2020). Thus, integration of various ‘omics’technologies (Fernie and Schauer, 2009; Weckwerth, 2011; Ghatak et al., 2017a; Ghatak et al., 2017b; Ghatak et al., 2018; Weckwerth et al., 2020) and genetic engineering approaches including RNAi and synthetic biology with advanced plant breeding tools including speed breeding, genomic selection, ‘integrated genomic–enviromic prediction,’ and genome editing tools could help develop grain legumes with high viral disease resistance to meet the rising demand for food supply.

## Author contributions

UJ and KS conceptualized the idea and wrote the manuscript. HN, MT, RB, AL, SG, GD, and PP wrote the genomics section. YN and AC drew the figures and wrote the transgenic section. All authors contributed to the article and approved the submitted version.
